# Editorial

**Published:** 1916-02

**Authors:** 


### EDITORIAL

The Atlanta Journal-Record of Medicine has its Business Office at 23 West Alabama Street where all business communications should be sent.

Remittances are payable to The Journal-Record of Medicine.

Concerning Original Communications and Editorial matters, address Richard R. Daly, M. D., 708 Empire Life Building, Atlanta, Ga.

Reprints of Original Articles will be furnished at cost price. Requests for them had best be made on the Manuscript.

Upon request, we will present, postpaid, to each contributor of an original article, twenty copies of The Journal-Record of Medicine containing such article.

### EDUCATIONAL DEVELOPMENT IN ATLANTA.

It is likely that the origin of the missionary spirit came with the desire to share things that a fulness of possession imparts to the normal man and when that wealth comprised information which was either discovered or acquired after hard labor the donors became the teachers and the most respected citizens in the community. They could not help telling things that were good for their fellows and they marked their progress of acquisition by setting up and maintaining greater facilities for teaching.

In this splendid city of the South there are treasures and funds of educational value that demand outlets and these in conjunction with the allied desires of men round about us have had their expression and are making their records in the development of great schools where all may be taught to all who will learn. The university idea has taken hold with a new grip and with a greater aspiration than ever before. Profiting by all that has been experienced throughout the land and inspired by the genious of wonderfully equipped men who are aided in turn by big and wealthy men who are appreciative of the time and opportunity, the universities of Atlanta are under way with the best of plans for construction and the broadest of ideas as to teaching that are to be obtained.

The people of the city have recognized the advances of education and are setting up the permanent landmarks marking an epoch in educational development. They sympathize with all that has been done and suggested and they contribute munificently of their means toward the construction of the buildings and the encouragement of the teachers whom they would trust and honor.

Emory University has been formed from the fine old College that has turned out a splendid alumni and in the reorganization of medical teaching the University has taken over the Medical College as one of its co-ordinate units. That great advantages will accrue to both is undoubted for each has the greater chance to be of service under the extended facilities.

Oglethorpe University and the Theological Seminary are progressing rapidly and the great hopes they have set before them have led them to a satisfactory point already.

These great plans rapidly nearing completion, have attracted country-wide attention and young men are seeking admission to the community that will afford them the opportunities they have dreamed of and longed for. The ideal climate of Atlanta adds its charm to university life and it welcomes all comers and proves its hospitality throughout the year. It will be a revelation to many Northern men to find that the warm summer months are pleasanter here than they are in many localities nearly a thousand miles to the north, and on this account it is expected that summer schools will ultimately prosper as part of the teaching plan.

Of course, it is in medical training that we are particularly interested and on this account the Emory University with its fine medical school attracts our attention and secures our cooperation.

In a future number there will appear an extended notice of this school so that our readers may be familiarized with the unusual opportunities this city will present to those seeking instruction in medicine, whether undergraduate or post graduate.

### OFFICE APPEARANCES.

The time is close at hand when the young man who has completed his college and hospital career will be making ready for his work and setting up his own office for the first time.

Very likely past expenditures are marked with due bills and financial obligations weigh upon him depressingly. Fortunate, indeed, is the man whose patrimony extends through this period and enables him to contemplate the waiting time without worry and to furnish his office correctly as a matter of investment.

We well know the fears and doubts of the young man who faces first a rental charge that is all outgo from the day he enters his office and threatens to his timid imagination a growth whose persistence is worse than a gastric carcinoma. We sympathize with him when agents of all sorts attempt to sell him apparatus and show him how indispensible each outfit is and what successes his rivals are having with the latest $250 machine or how he can not expect to keep up with the times unless he has this encyclopaedia or that set of journals. We understand his dismay when the plumber says that such a thing will cost him more than he expected to spend for his meals during the first thre months and the painter presents sign painting bills for enough to outdo even the printer who has made him mortgage his room rent for stationery.

The medical society dues loom large, the social gathering expenses incite an agony of suspense and the laundry man seems to be a potential ogre who may never return with the shirts that link the young man to gentility unless more money is forthcoming from an unknown source: yet all these simply must be attended to and with them there is the great duty of making a good showing. The smile a young man carries rests easily upon him and its very suggestiveness makes it seem real and a happy mood comes with the elastic temperament which is the man's best stock in trade after his education.

But there is no use in bragging about making expenses the first month and carrying the bluff around among friends and strangers who may chance to be listening on the street cars when one can not back it up with something. If the backing can be shown, the bluff holds good a long time and will weather a man through until he can pass it along to some other needy chap.

The best backing a beginner can have when he is starting by himself is a well-equipped and prosperous appearing, comfortable and clean office. Nothing will give a patient or visitor a sharper impression of a man's attitude toward life than a tasteful and substantial waiting room where his visitor will be welcomed, and then a consultation room that is correct. There is no greater handicap than make-shift furnishings in either place and the correct ones should be secured at any sacrifice a gentleman can make. When one thinks of all the years of preparation he has spent in order to go to the public on his own hook, he should never let go the idea that he must meet the public as he wants the public to meet him. His office should show cleanliness, correctness, simplicity and dignity in all its furnishings from the very beginning. The reaction of these will be potent upon not only the visitors but upon himself. That is about the only kind of backing and about the only approximation to bluff that we know of as successful in medicine and this works only to replace timidity with a normal self-confidence.

